# Magnetic resonance microscopy and correlative histopathology of the infarcted heart

**DOI:** 10.1038/s41598-019-56436-5

**Published:** 2019-12-27

**Authors:** Itziar Perez-Terol, Cesar Rios-Navarro, Elena de Dios, Jose M. Morales, Jose Gavara, Nerea Perez-Sole, Ana Diaz, Gema Minana, Remedios Segura-Sabater, Clara Bonanad, Antoni Bayés-Genis, Oliver Husser, Jose V. Monmeneu, Maria P. Lopez-Lereu, Julio Nunez, Francisco J. Chorro, Amparo Ruiz-Sauri, Vicente Bodi, Daniel Monleon

**Affiliations:** 1 Laboratory of Metabolomics, Institute of Health Research-INCLIVA, Valencia, Spain; 2 Department of Cardiology, Hospital Clínico Universitario, INCLIVA, Valencia, Spain; 3 Unidad Central de Investigación Biomédica, University of Valencia, Valencia, Spain; 4 Pathology Department, School of Medicine, University of Valencia, Valencia, Spain; 5 Centro de Investigación Biomédica en Red – Cardiovascular (CIBER-CV), Madrid, Spain; 6 Medicine Department, School of Medicine, University of Valencia, Valencia, Spain; 7 Cardiology Department and Heart Failure Unit, Hospital Universitari Germans Trias i Pujol. Department of Medicine, Universitat Autònoma de Barcelona, Barcelona, Spain; 8 Department of Cardiology, St.-Johannes-Hospital, Dortmund, Germany; 9 Cardiovascular Magnetic Resonance Unit, ERESA, Valencia, Spain; 10 Centro de Investigación Biomédica en Red – Fragilidad y Envejecimiento Saludable (CIBER-FES), Madrid, Spain

**Keywords:** Cardiology, Diagnostic markers

## Abstract

Delayed enhancement cardiovascular magnetic resonance (MR) is the gold-standard for non-invasive assessment after myocardial infarction (MI). MR microscopy (MRM) provides a level of detail comparable to the macro objective of light microscopy. We used MRM and correlative histopathology to identify infarct and remote tissue in contrast agent-free multi-sequence MRM in swine MI hearts. One control group (n = 3 swine) and two experimental MI groups were formed: 90 min of ischemia followed by 1 week (acute MI = 6 swine) or 1 month (chronic MI = 5 swine) reperfusion. Representative samples of each heart were analysed by contrast agent-free multi-sequence (T1-weighting, T2-weighting, T2*-weighting, T2-mapping, and T2*-mapping). MRM was performed in a 14-Tesla vertical axis imager (Bruker-AVANCE 600 system). Images from MRM and the corresponding histopathological stained samples revealed differences in signal intensities between infarct and remote areas in both MI groups (p-value < 0.001). The multivariable models allowed us to precisely classify regions of interest (acute MI: specificity 92% and sensitivity 80%; chronic MI: specificity 100% and sensitivity 98%). Probabilistic maps based on MRM images clearly delineated the infarcted regions. As a proof of concept, these results illustrate the potential of MRM with correlative histopathology as a platform for exploring novel contrast agent-free MR biomarkers after MI.

## Introduction

Delayed enhancement cardiovascular magnetic resonance (MR) is the most used imaging technique for comprehensive non-invasive assessment of the structural consequences of myocardial infarction (MI) in patients^1^. However, limitations to the use of contrast in cardiovascular MR include the risk of side effects and the length of the studies^2^. Therefore, it seems important to advance towards contrast agent-free, histologically-validated cardiovascular MR biomarkers.

Each sequence of a cardiac MR study provides specific information, which when taken together provides a comprehensive picture of the cardiac structure. T1-weighted images show the cardiac anatomic structure, T1 values rise with increased extracellular volume, while T2-weighted images are related with the magnitude of the myocardial oedema^3,4^. Novel sequences recently incorporated into the clinical armamentarium are designed to indirectly evaluate fibrotic tissue (T1-post contrast and extracellular volume fraction), the presence of oedema during the acute post-MI phase (T1-mapping^5,6^), and the extent of haemorrhage in the infarcted area (T2*-mapping^2^). Despite the benefits provided by endogenous contrast in T1, T2, and T2* images and the design of new sequences, gadolinium-enhanced cardiac MR remains the routine tool in Post-Infarction imaging. Its high tissue contrast makes it the method of choice to measure infarct size and determine the transmural extent of infarction^7,8^.

The term MR microscopy (MRM) refers to ultra-high resolution (<100 µm) MR imaging. This resolution is lower than that of light microscopy (0.25 µm), but much higher than clinical MR (approximately 1 mm in plane resolution)^9^. Thus, MRM provides a more detailed functional and anatomical picture of tissue than clinical MR. In addition, ultra-high resolution technology is becoming more accessible to clinics, and some studies have shown promising results on the utility of cardiac images from a 7T scanner^10,11^. Histopathological validation could act as a bridge between MRM and clinical MR^12–14^ and help develop novel contrast agent-free cardiovascular MR biomarkers in the field of myocardial diseases.

The end-point of the present study was to illustrate the potential of contrast agent-free MRM and correlative histopathology as a source of future cardiovascular MR biomarkers. For this purpose, and as a proof of concept, we tested the accuracy of this methodology in highly-controlled swine models of acute and chronic MI to identify the most characteristic structural consequence in this scenario, namely the presence of infarcted tissue. The specific objectives were: (1) to distinguish between infarcted and remote areas (in the acute and chronic MI settings) by contrast agent-free MRM sequences, identified and validated by correlative histopathology. (2) To analyse the value of combined MRM parameters using multivariate discrimination models for the same purpose. (3) To represent the information provided by the merged multivariate models in pixel-by-pixel probabilistic maps for intuitive tissue classification and easier interpretation.

## Results

One control group (n = 3 swine) and two experimental MI groups (one acute and other chronic) were formed: 90 min of ischemia were followed by 1 week (acute MI, n = 6 swine) or 1 month (chronic MI, n = 5 swine) reperfusion.

**Figure 1:**
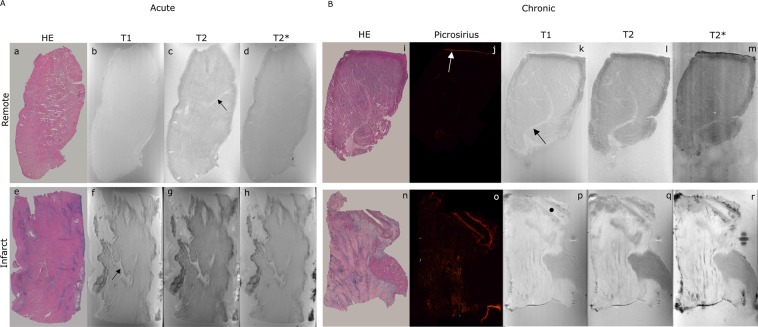
Histopathological sections and magnetic resonance microscopy images of an acute and a chronic MI experiment. Left panel showing acute MI images (**A**): Histopathological stain with haematoxylin-eosin (a,e), T1-weighted imaging (b,f), T2-weighted imaging (c,g) and T2*-weighted image (d,h) for remote and infarcted tissues respectively. Right panel showing chronic MI images (**B**): Histopathological stains with HE (i,n) and picrosirius red (j,o), T1-weighted imaging (k,p), T2-weighted imaging (l,q) and T2*-weighted imaging (m,r) for remote and infarcted tissues respectively. Connective tissue is marked with a black arrow, pericardium is marked with a white arrow and a vessel is marked with a black dot. Areas with and without necrosis (**A**) or fibrosis (**B**) (shown by histopathology) can be easily distinguished in terms of image intensity by magnetic resonance microscopy. Abbreviations: HE = haematoxylin-eosin; MI = myocardial infarction; T1 = longitudinal relaxation; T2 = transversal relaxation; T2* = effective transversal relaxation.

Briefly, following heart excision, infarct and remote myocardial samples from each heart were isolated and measured in the 14 T MRM equipment. These same samples were then histologically stained to identify remote and infarct areas. The images obtained from the histology were then compared with those derived from MRM in order to identify the remote and infarct areas in the MRM images (Fig. 1).

### MR microscopy of the acute and chronic MI groups

#### Acute MI group

In comparison to the uniform signal intensity of the remote tissue, the great heterogeneity in signal intensity shown by infarcted tissue in T1, T2, and T2* MRM images allowed us to clearly differentiate between both tissue types (Fig. 1A).

**Figure 2:**
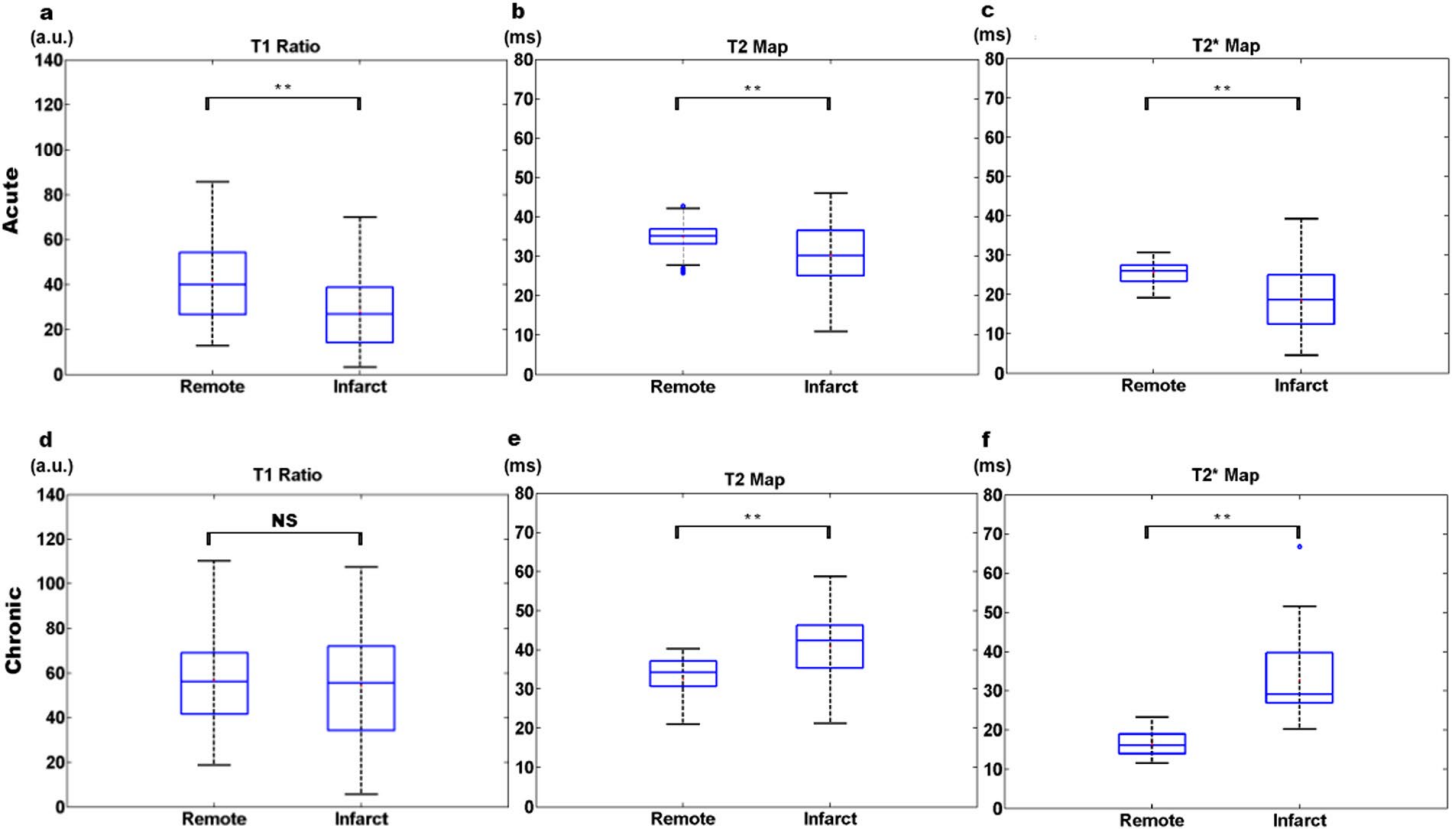
Boxplots representing magnetic resonance microscopy parameters in acute and chronic MI experiments. Box-and-whisker plots showing T1 signal intensity ratio (**a,d**), T2 map (**b,e**), and T2* map (**c,f**) for remote and infarct areas in acute (upper panel) and chronic (lower panel) MI hearts. Boxes denote interquartile range, lines denote median, and whiskers denote tenth and ninetieth percentiles. The statistical significance is shown for each sequence between infarct and remote tissue (**p < 0.01). Abbreviations: au = arbitrary units; MI = myocardial infarction; T1 = longitudinal relaxation; T2 = transversal relaxation; T2* = effective transversal relaxation.

In the acute MI, T2 and T2* values from infarct areas were lower and with larger standard deviation in comparison to remote tissue (Fig. 2 upper panel). This reflects that the acute infarcted tissue is more active, and different process (oedema, infiltration and necrotic tissues) are occurring at those moments. The high inter- and intra-sample heterogeneity in acute MI samples is due to the heterogeneous composition of the infarcted region.

In the infarcted area, the histopathological examination identified oedema, inflammatory infiltrate and necrosis (Fig. S1). Haematoxylin staining highlighted areas of leucocyte accumulation, typical of the inflammatory infiltrate. And eosin staining revealed the accumulated inner liquid of the cytoplasm, as well as the necrotic cells, that has lost the limits. In T2-weighted imaging, regions with severe inflammatory infiltrate showed as dark spotted areas. Oedematous areas appeared grey, and necrotic areas displayed bright tones in all three sequences (Fig. 1A).

ANOVA analysis was used to compare remote, infarct and control for T1 signal intensity values, T2 map values, and T2* map values. Differences for control-remote, control-infarct and remote-infarct were all statistically significant (p < 0.05) (Table S1).

#### Chronic MI group

An excellent correlation existed between histopathological staining and MRM images derived from T1, T2 and T2* weighted images (Fig. 1B) in this setting. Under MRM, infarcted areas appeared predominantly hyper-intense with hypo-intense splashes. This is indicative of tissue damage repair by a collagen scar.

This pathological evolution resulted in reversed quantified values of T2 and T2* for chronic with respect to acute infarcted samples, reflecting the replacement of oedema and inflammatory infiltration by collagen deposition in chronic MI samples. The remote tissue exhibited low intra- and inter-sample heterogeneity and a higher standard deviation than the remote tissue from the acute model. Moreover, the values of all three sequences from the infarcted tissue of the chronic model increased substantially due to the formation of collagen scar (Fig. 2 lower panel).

For this group, we also compared infarct, remote and control areas for each of the three sequences. As in the acute MI group, differences for control-remote, control-infarct and remote-infarct areas were highly significant (p < 0.001) (Table S1).

### Multimodal analysis to differentiate between infarcted and remote regions

#### Acute MI model

**Figure 3:**
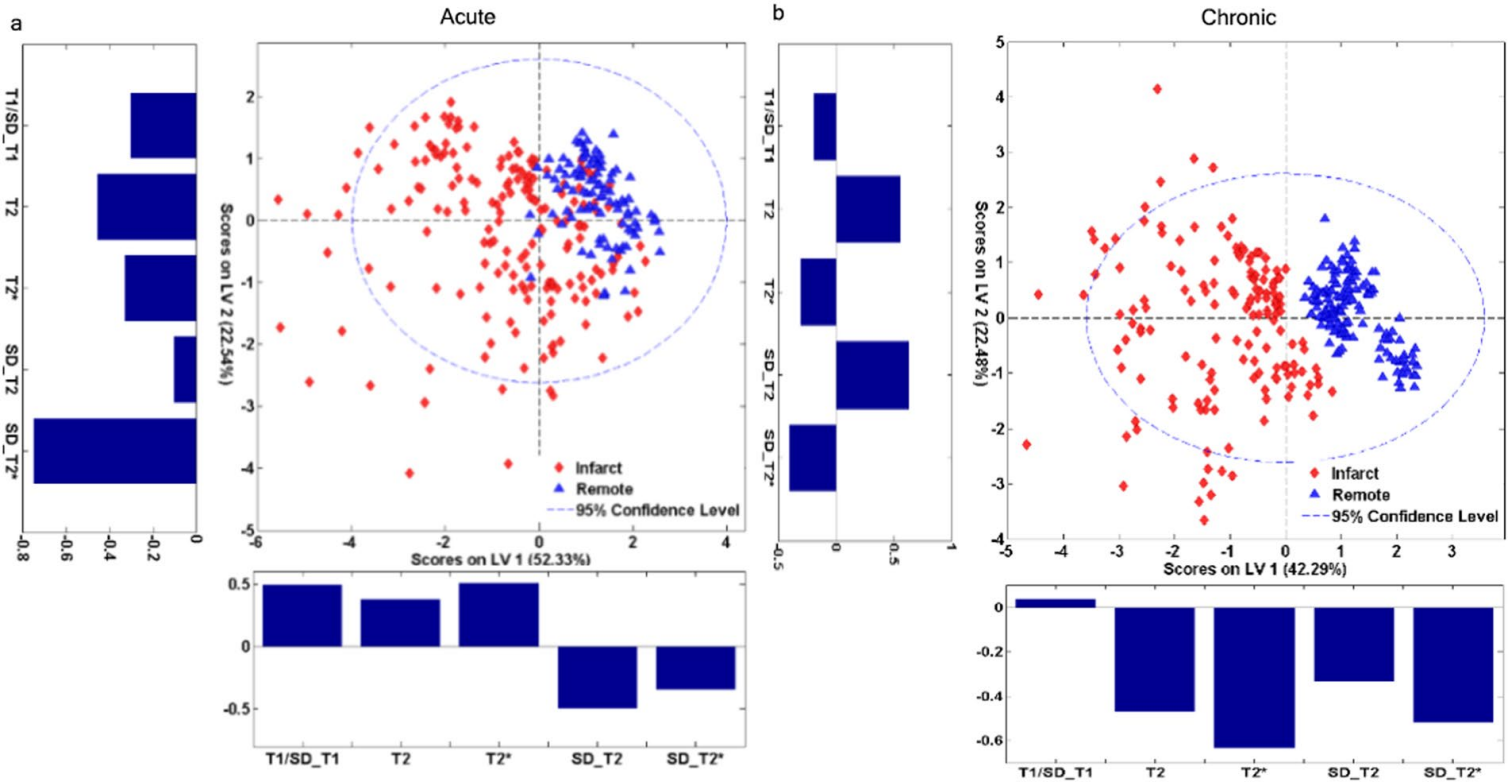
Scores and loading plots of the PLS-DA models to differentiate between the infarct and the remote areas in magnetic resonance microscopy images of acute and chronic MI samples. Axes correspond to latent variables that explain more than 50% of data variability. Both the acute (**a**) and chronic (**b**) models were able to discriminate between the infarct (red points) and the remote (blue) areas. The merged PLS-DA model for chronic MI permitted an excellent differentiation between remote and infarcted tissue. Abbreviations: LV = latent variable; MI = myocardial infarction; PLS-DA = partial least squares discriminant analysis; SD = standard deviation; T1 = longitudinal relaxation; T2 = transversal relaxation; T2* = effective transversal relaxation.

Cross validation error rate for the acute infarct model was 15.71% (44/280). Specificity and sensitivity of this model in correctly identifying infarct and remote regions of interest (ROIs) were 0.92 (92/100) and 0.80 (144/180), respectively. Root mean square cross validation error was 0.52 for the remote tissue and 0.75 for the infarcted tissue. In the acute infarct partial least squares discriminant analysis (PLS-DA) model, the latent variable (LV) 1 (X axis) explained 52.33% of dataset variance. The loading plot for this LV1 revealed that T1 ratio, T2* mean, and T2 standard deviation contributed the most to the direction of discrimination. The LV2 (Y axis) explained 22.54% of dataset variance. T2* standard deviation contributed the most to LV2 according to its loading plot (Fig. 3a).

#### Chronic MI model

Cross validation error rate for the chronic infarct model was 0.69% (2/290). This indicates that the merged multi-sequence model permits an excellent discrimination between the infarcted and the remote tissue in the chronic MI model. The specificity and sensitivity of the chronic MI PLS-DA model in correctly identifying ROIs sampled from the infarcted area were 1.00 (140/140) and 0.99 (148/150), respectively. Root mean square cross validation error in chronic infarct samples was 0.57 for the remote tissue and 0.60 for the infarcted tissue.

In the chronic MI PLS-DA model, the LV1 explained 42.29% of dataset variance and perfectly differentiated between the infarcted and the remote tissue (X axis). The loading plot for this LV1 revealed that T2* with its standard deviation contributed the most to this axis. The LV2 explained 22.48% of dataset variance (Y axis). T2 and its standard deviation contributed the most to LV2 according to the loading plots (Fig. 3b).

### Probabilistic images

Thus, once the T1 virtual variable (T1 signal intensity mean/T1 signal intensity sd ratio) and, T2 and T2* relaxation times mean and its standard deviation matrixes were combined in multi-sequence approaches, the resulting PLS-DA multivariate models for acute and chronic MI discriminated between infarcted and remote tissue with high specificity and sensitivity. We built probabilistic images to easily visualize these results.

**Figure 4:**
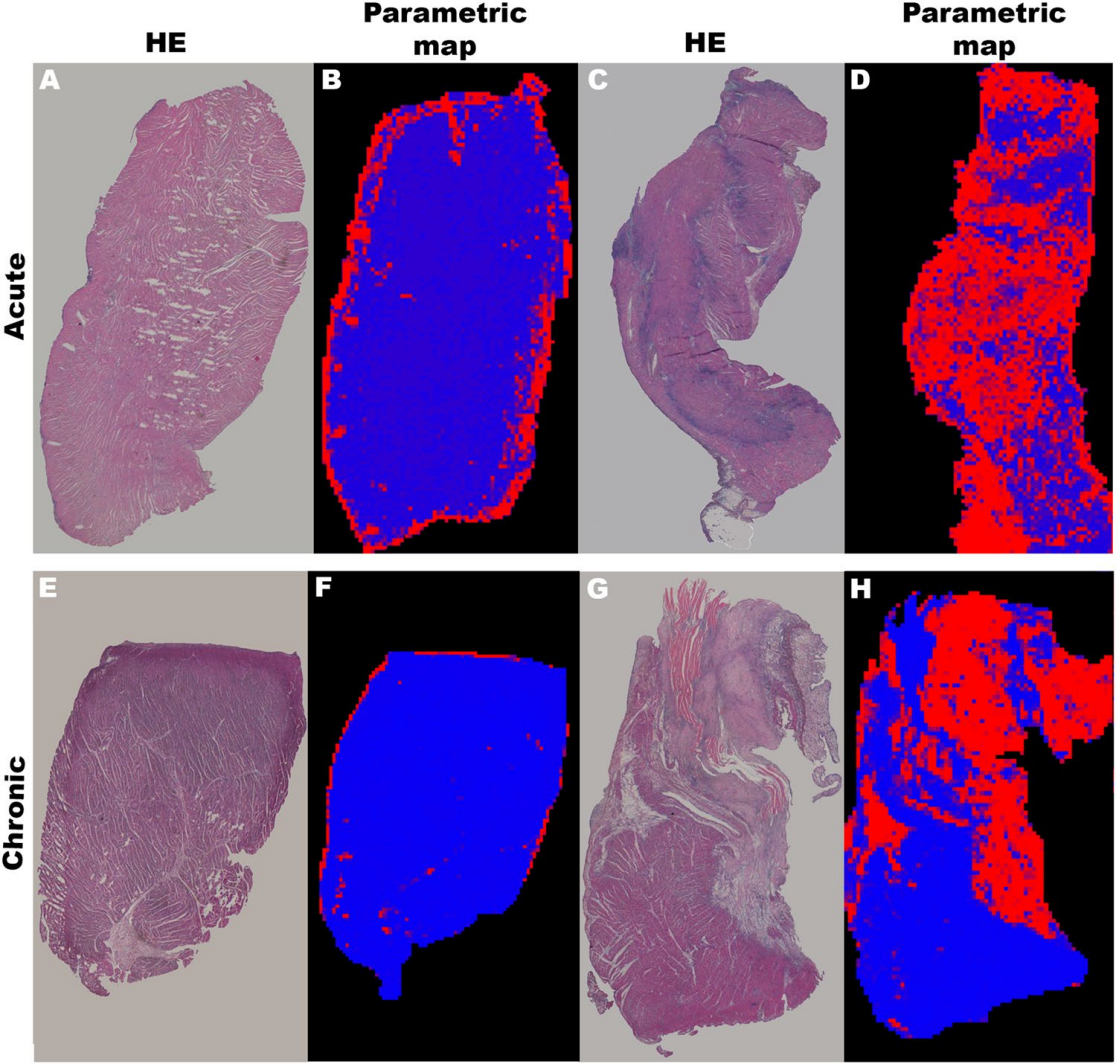
Magnetic resonance microscopy probabilistic maps of myocardial samples obtained from swine infarcted hearts. The probabilistic maps used a red-blue colour code to represent the combined probability (based on the respective PLS-DA models) of the tissue being infarcted (red) or remote (blue). The correlative histopathology is also shown. Remote areas (acute infarction: **A,B**; chronic infarction: **E,F**) and infarct areas (acute infarction: **C,D**; chronic infarction: **G,H**) are displayed in the left and right panels respectively. An agarose-hiding mask has been applied to the map to better visualize images. Abbreviations: HE = haematoxylin-eosin; PLS-DA = partial least squares discriminant analysis.

The probability of a given ROI, based on the merged PLS-DA models, of belonging to either the infarcted or remote tissue was graphically represented: red for infarcted tissue and blue for remote tissue. Figure 4 displays the probabilistic maps for samples obtained from the remote and the infarcted tissue in both the acute and chronic MI models.

## Discussion

The main contribution of the present study is that it illustrates the possibilities of contrast agent-free multisequence MRM with correlative histopathology as a platform to investigate and develop new MR biomarkers in the field of myocardial diseases with a potential for translation into the clinic. As a proof of concept, our results demonstrate that this approach permits an accurate identification of the most relevant features of myocardial damage following MI, namely the magnitude of necrotic (in acute phase) and fibrotic (in chronic phase) tissue. We have previously used this approach in the evaluation of brain tumours^15^, but to our knowledge this is the first study in myocardial samples. The methodology applied in the present study could form the basis for future research aimed at investigating new contrast agent-free cardiovascular MR biomarkers in myocardial diseases.

The use of a 14 T scanner with a spatial resolution higher than conventional MR permitted the visualization of small myocardial samples in high microstructural detail. Imaging was carried out with conventional cardiac MR sequences (T1-weighted, T2 and T2* maps)^7^ and samples were embedded in an agarose matrix for subsequent matching of MRM images with light microscopy. Histopathology by optical microscopy ensured the correct interpretation of the different MRM images and a better identification of the microstructural elements present in the samples. At 14 T, without an additional contrast agent, the images were sufficiently detailed to identify the major structural components of the tissue. For direct co-registration of MRM and histopathology, we requested both histological and MRM virtual slices in the plane of maximal area. As shown in Fig. 1, MRM had a similar resolution to histology of the macro objective of the light microscope. Correlating MRM images with histopathology was key to identifying microstructural properties in specific regions of interest defined by the histologist.

MRM quantification revealed differences in T2 and T2* values between chronic and acute infarct samples. Haematoxylin-eosin staining revealed a heterogeneous tissue composition (oedema, necrosis, and inflammatory infiltrate) in the acute phase^2^, which may explain the high intra- and inter-sample heterogeneity detected in the acute infarct model. In contrast, as found in previous studies^16–18^, in chronic infarct samples oedema had already vanished and a homogeneous scar with high collagen content (as shown from picrosirius red stain) had substituted most of the damaged tissue. This explains why T2 and T2* were much more consistent in the chronic MI model.

Although each individual MRM sequence displayed some microstructural elements, only the combined multi-sequence analyses provided specific characteristic patterns that revealed pathologically-relevant structures. Other studies performed with both visual and quantitative MR have shown that it is not always possible to distinguish between infarcted and remote regions in a single sequence^19^. In the present study, the use of PLS-DA multivariable models to simultaneously analyse multi-sequence MRM images and extract the most valuable data from each was decisive in precisely differentiating between infarcted and remote areas.

Our findings reveal that not only image intensity, but also intra- and inter-region variability, expressed as standard deviations, contribute decisively to discrimination. Thus, beyond changes in the nature of the tissue, the high degree of heterogeneity present in the infarcted myocardium (especially in acute phase) alters its functional properties and must also be taken into account in detection. This accurate discrimination probably reflects the higher homogeneity exhibited by the chronic infarcted tissue when compared with the unstable condition and heterogeneous composition of the infarcted tissue in the acute MI group. The almost perfect accuracy (99% sensitivity, 100% specificity) of the PLS-DA model in detecting fibrotic areas in the chronic MI group reflects the virtues of the presented methodology.

Expert operators are accustomed to interpreting MR images. However, the simultaneous use of several images and sequences over the same anatomical regions is often a cumbersome task and subjective^4^. Combining the mathematical data derived from PLS-DA models to provide a simple probabilistic view represents a user-friendly approach that facilitates the analysis for MR operators. By combining all sequences used in a 3D matrix and running each pixel through the PLS-DA model, we obtain a pixel-by-pixel probability matrix that expresses the probability of each pixel belonging to remote or infarct tissue. The conversion of this information into a 3D matrix classifies the different regions of the infarcted heart with a pixel-by-pixel RGB code and generates easy-to-interpret parametric maps. The similarity between our probabilistic images and the histological evaluation of the same samples illustrates the reliability of this approach.

In routine cardiovascular MR, late gadolinium enhancement permits a reliable quantification of necrotic and fibrotic tissue after MI. As mentioned above, the use of gadolinium contrast can exert deleterious effects on patients with poor kidney function, prolong studies and reduce lab efficiency. Recently, contrast agent-free T1ρ^20,21^, native T1 map^6,22,23^ and diffusion-weighted^24^ imaging have been incorporated for this purpose. The present study does not aim to substitute these well-validated indexes.

Our results illustrate the potential of MRM using multivariable methodology applied in a 14 T field without additional contrast to properly identify the major structural components of myocardial tissue indicated and confirmed by histopathology. This approach, tested in the present study as a proof of concept to detect necrotic and fibrotic tissue in experimental acute and chronic swine MI models, could be used in future as a platform to generate an *in-vivo* model for obtaining new potential cardiovascular MR biomarkers that could be translated into the clinic in the field of myocardial diseases.

The translation of this approach into clinical field strengths will be complex but is feasible. Generation of the MR signal is based on the same physical principles regardless of field strength. Although important limitations prevent direct translation (removed blood effects, main magnetic field, B_0_ inhomogeneities, partial tissue degradation, etc.), the described methodology can reveal MR parameters with potential as clinical MR biomarkers. Although these potential biomarkers suffer from many limitations they could help direct future clinical MR biomarker research into more meaningful directions and aid in the interpretation in terms of histopathological events. The final clinical biomarker development and validation, based on high-field findings, should be performed in clinical scanners.

Our study suffers the inherent limitations of studies performed on experimental samples that advise caution on the translation of such findings to clinical MR imaging. For example, the physical and physiological conditions of the tissue directly affect the MRM parameters. The data herein presented are a first step to show the potential of MRM and correlative histopathology as a source of new cardiovascular MR biomarkers and as a platform to improve the pathological understanding of cardiovascular MR findings after MI.

## Materials and Methods

### Study in the experimental swine model of reperfused MI

The study was approved by the Animal Care and Use Committee of the University of Valencia and conformed to the Guide for the Care and Use of Laboratory Animals published by the U.S. National Institutes of Health (NIH Publication No. 85–23, revised 1996) and to The ARRIVE guidelines (www.nc3rs.org.uk/ARRIVE).

#### Experimental protocol

Well established experimental swine models of acute and chronic MI were used. Experiments were coordinated by the veterinarian of the University of Valencia, who has participated for more than 10 years in the development of our swine infarction model. After fasting for at least 12 h, juvenile domestic pigs weighing 25–30 kg were sedated using intra-muscular 8 mg kg^−1^ ketamine and 0.1 mg kg^−1^ medetomidine, and anesthetized using 10 mg kg^−1^ h^−1^ continuous intra-venous infusion of 2% propofol. The right jugular vein was cannulised to administer drugs and to obtain blood samples. Before the intervention, intra-venous amiodarone (300 mg) and lidocaine (30 mg) were administered to the pigs to diminish life-threatening arrhythmias, and heparin (3000 U) to diminish the formation of thrombus. Animals were mechanically ventilated using a 50% oxygen gas mixture and continuously monitored through electrocardiogram for heart rate, rhythm, and ST-segment changes. An interventional cardiologist with 9 years’ experience performed cardiac catheterizations.

A 7F sheath was introduced into the right femoral artery to monitor blood pressure and to access the left anterior descending coronary artery. A 7F Amplatz Left 0.75 catheter was used to selectively engage the left coronary artery, and a standard hydrophilic angioplasty wire was advanced and placed in the left distal anterior descending coronary artery. A 2.5 × 15 mm over-the-wire angioplasty balloon was inflated to 6 atm immediately distal to the first diagonal branch and coronary artery occlusion was confirmed by contrast injection and by electrocardiographic ST-segment elevation. Additional details on our experimental models can be consulted elsewhere^14,25,26^.

#### Experimental groups

One control group and two MI (acute and chronic) experimental groups were formed. In the MI groups, after 90-min occlusion of the mid-left anterior descending artery by an angioplasty balloon, experiments were categorized as follows: (1) 1-week (n = 6, acute MI) and (2) 1-month (n = 5, chronic MI) reperfusion. The control group (n = 3) was subjected to the same experimental protocol used in the MI groups, but without angioplasty balloon inflation, thus ischemia and infarction were not induced.

#### Macroscopic study of the infarct and remote regions

Once the reperfusion period was completed (1 week in acute MI, 1 month in chronic MI), cardiac catheterization was repeated under the same conditions used in the first procedure. In order to determine the area at risk, 20 mL of 4% thioflavin-S (TS) (Sigma Aldrich, St Louis, MO, USA) solution were injected distally into the coronary artery through the lumen of the over-the-wire balloon inflated at the same point and to the same pressure as that used to induce MI. Finally, the heart was arrested with potassium chloride and excised in all cases^14,25,26^.

**Figure 5:**
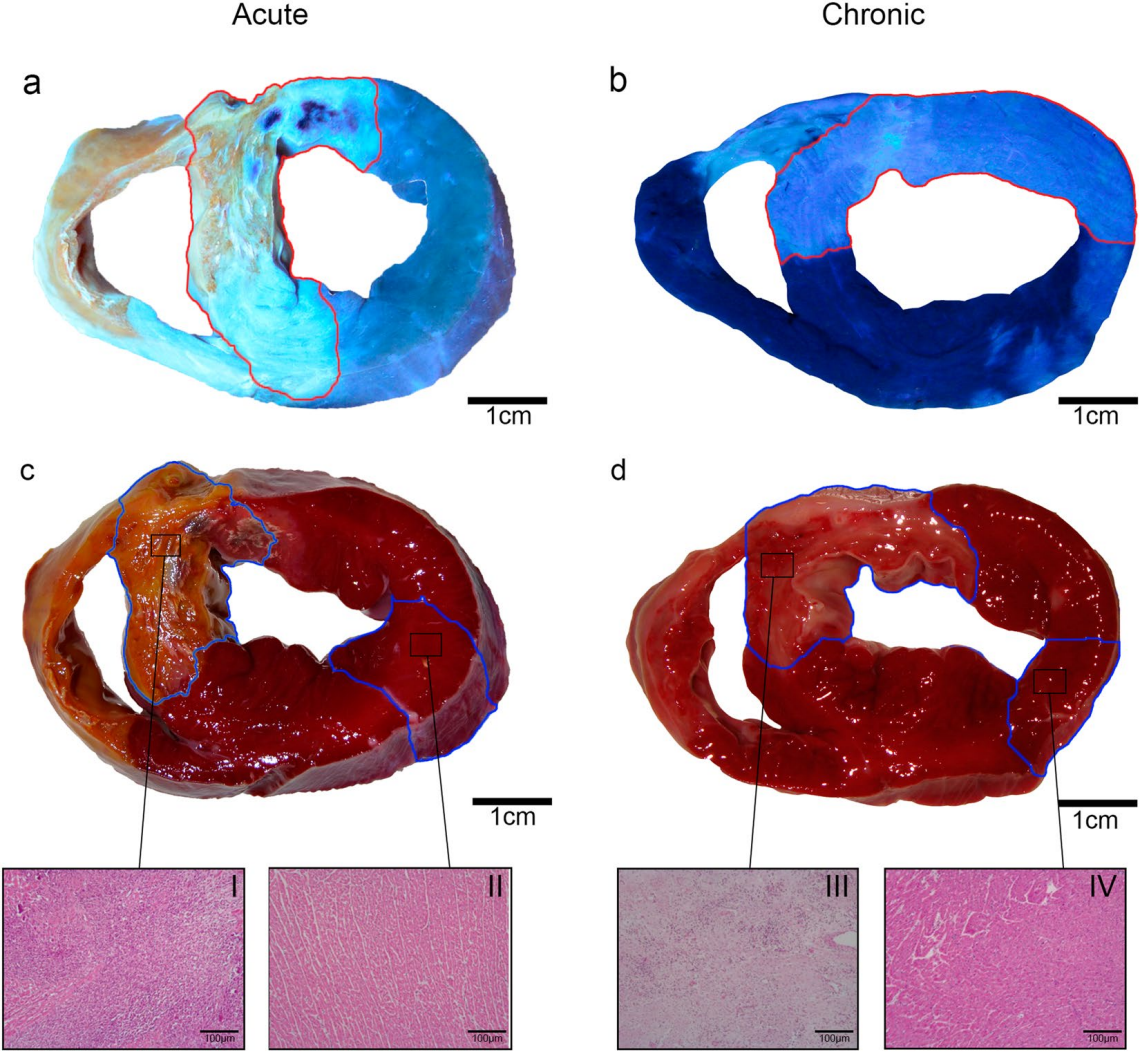
Macroscopic heart sections from the acute (left panels) and chronic (right panels) myocardial infarction groups. Upper panels (**a,b**): Area at risk corresponds to tissue stained with thioflavin-S (light blue). Lower panels (**c,d**): The infarcted tissue corresponds to 2,3,5-Triphenyltetrazolium non-stained myocardium. Samples were obtained from the acute (c: I-infarct, II-remote) and chronic infarct models (d: III-infarct and IV-remote) and stained with haematoxylin-eosin.

The left ventricle of the excised heart was sectioned into 5-mm thick short-axis slices. Pieces were incubated in 2% 2,3,5-triphenyltetrazolium chloride (TTZ) solution (Sigma Aldrich, St Louis, MO, USA) at 37 °C for 20 minutes to determine the infarcted area. The infarcted area was defined as myocardium with simultaneous TS (within the area at risk) and without TTZ uptake, whereas myocardium with TTZ uptake (non-infarcted) and without TS uptake (out of the area at risk) was considered as remote myocardium (Fig. 5)^14,25,26^.

### Sample preparation for MRI

Samples, each approximately 1 × 1 × 0.50 cm^3^, were taken from the infarcted and remote areas in each of the animals. Overall, 36 myocardial samples were obtained and classified into 3 groups: infarcted myocardium (7 chronic and 9 acute), remote myocardium (7 chronic and 5 acute) and controls (8 samples). Samples were preserved at −80 °C until further measurement.

### Magnetic resonance microscopy

Immediately before MRM acquisition, samples were embedded in a 2.5% agarose matrix (Agarose Low EEO, Scharlau, Barcelona, Spain) and placed inside a Ø 1 cm nuclear MR tube. MRM images were acquired at room temperature (25 °C) in a 14 T vertical axis imager (Bruker-AVANCE 600 system, Bruker Biospin GmbH, Rheinstetten, Germany) equipped with a 10 mm microimaging ^1^H coil tuned to the appropriate proton frequency. The acquisition software was ParaVision 4 (Bruker Biospin GmbH, Ettlingen, Germany).

Slice orientation was selected to provide the largest in-plane tissue area. Maximal gradient strength was 210 gauss cm^−1^. Matrix resolution was 256 × 256 pixels. The field of view was adjusted for each sample to improve the resolution. Slice thickness was 0.50 mm without gap. Our standard protocol for imaging lasted 9 hours and consisted of rapid acquisition with refocusing echo sequences to obtain T1- and T2-weighted imaging with the following repetition time and echo time (TR/TE) values: T1-weighted imaging, 1500 ms/9.3 ms and excitation flip angle 90°; T2-weighted imaging: 4000 ms/38.4 ms and excitation flip angle 90°. The acquisition bandwidth for the T1 experiment was set to 3.6 kHz and 128 signal averages were acquired, leading to a total scan time of 2 hours, 33 minutes and 36 seconds. The acquisition bandwidth for the T2 experiment was set to 2.7 kHz and 64 signal averages were acquired, leading to a total scan time of 3 hours, 24 minutes and 48 seconds. Multi gradient echo pulse sequences were also acquired to obtain T2*-weighted imaging with TR/TE values of 2000 ms/9 ms and excitation flip angle 30°. The acquisition bandwidth for the T2* experiment was set to 5.4 kHz and 8 signal average was acquired, leading to a total scan time of 51 minutes and 12 seconds. We also performed multi slice, multi echo pulse sequences to T2 mapping (TR of 2500 ms and 16 echos ranging from 9.8 to 156.4 ms, excitation flip angle of 90°) and multi gradient echo pulse sequences to T2* mapping (TR of 2000 ms and 12 echos starting at 4 ms with 6 ms intervals, excitation flip angle of 30°). The acquisition bandwidth for the T2- mapping experiment was set to 3 kHz and 16 signal averages were acquired, leading to a total scan time of 2 hours and 8 minutes. The acquisition bandwidth for the T2*- mapping experiment was set to 5.4 kHz and 8 signal averages were acquired, leading to a total scan time of 51 minutes and 12 seconds. This measurement system allows an almost free choice of virtual slice selection and orientation. The maps were calculated with the t2vtr equation. Depending on the sequence used, in-plane spatial resolutions achieved up to 35 µm per pixel. Table 1 shows the MR sequences parameters used.

**Table 1:** Parameters of the magnetic resonance microscopy sequences employed.

SEQUENCES	TR (ms)	TE (ms)	Echoes	Averages	RF	Flip Angle	Matrix	Measurement method	Slices number
T1-weighted imaging	1500	9.3	1	128	4	90°	256 × 256	RARE	12
T2-weighted imaging	4000	38.4	1	64	4	90°	256 × 256	RARE	12
T2*-weighted imaging	2000	9	1	8	—	30°	256 × 256	MGE	12
T2- multiecho sequences	2500	From 9.8 to 156	16	16	—	90°	256 × 256	MSME	12
T2*- multiecho sequences	2000	From 4 to 70	12	8	—	30°	256 × 256	MGE	12

Abbreviations: MGE = multi gradient echo sequence; MSME = multi slice, multi echo pulse sequence; RARE = rapid acquisition with refocusing echo sequence; RF = RARE factor; T1 = longitudinal relaxation; T2 = transversal relaxation; T2* = effective transversal relaxation; TE = echo time; TR = repetition time.

The quality of the images from each sequence was evaluated by quantifying the signal-noise-ratio for the chronic (Table S2) and acute (Table S3) MI groups. To calculate the signal-noise-ratio, the following formula was employed: ROI_signal_ mean/ROI_noise_ SD^27,28^.

### Histopathological analysis

After MRM, the agarose matrix was removed and the sample rinsed in a 4% formaldehyde solution (Formol 4% Buffered, VWR, Radnor, PA, USA). The sample was then embedded in paraffin and sliced (5 µm thickness) using a Leica RM2145 microtome (Leica Microsystems, Weztlar, Germany). Histological slicing orientation was chosen to match the MRM images following the maximal sample area criteria.

Characterization of histological slices was performed using conventional haematoxylin-eosin stain. Haematoxylin-eosin is the most used staining in medical diagnosis. Haematoxylin stains cell nuclei blue, and eosin stains the cytoplasms and extracellular matrix pink.

Additionally, in samples obtained from chronic MI experiments, the presence and distribution of collagen fibres were assessed using picrosirius red stain^26^. An optical microscope at 20x magnification (Leica DMD 108, Leica Microsystems, Weztlar, Germany) was used to capture images of all samples. A polarizing filter was applied to slices stained with picrosirius red (Direct Red 80, Sigma Aldrich, St Louis, MO, USA).

### Data analysis, chemometric classification models and probabilistic images

Based on histopathology, we first established that MRM sequences accurately discriminate between infarct and remote regions in both MI group (acute and chronic) images. The information provided by each MR sequence was combined to create a PLS-DA model for both MI groups (acute and chronic). We aimed to evaluate the accuracy of the merged multi-sequence models to properly distinguish between ROI obtained from the infarcted and remote areas.

In histopathological and in MRM slices, we identified common anatomical reference points to confirm location^14^. In each slice, we defined 10 square ROIs of 0.01 cm^2^ each. For samples from acute MI experiments, we selected 100 ROIs of remote tissue and 180 ROIs of infarcted tissue. We selected 160 ROIs from control tissue. For samples from chronic MI experiments, we selected 140 ROIs of remote tissue and 150 ROIs of infarcted tissue. Dataset was transferred into the mathematical environment Matlab R2013a (8.1.0.604 Natick, MA, USA). Statistical significance was tested using ANOVA test (p-value < 0.05) (Table S1).

For each ROI we calculated the mean and the standard deviation of T1-weighted intensities, T2 values, and T2* values. In order to quantitatively compare the homogeneity of T1 images between samples, we calculated the ratio between T1-weighted intensity mean and the corresponding standard deviation.

We then built PLS-DA models to differentiate between ROI groups (infarcted or remote) that included T1 ratio, T2 mean, T2 standard deviation, T2* mean and T2* standard deviation. PLS toolbox v8.0.2 (Eigenvector research, Inc., Manson, WS, USA) was used for this purpose. PLS-DA is a linear classification method based on the partial least squares regression algorithm. It is used to build a quantitative model for classification by projecting the predicted and observable variables into a new space to maximize the covariance between the response and independent variables. PLS-DA suggested using the first two LVs. The models were cross-validated using the venetian blinds approach. Receiver-operating characteristic curves were calculated for cross validation. Finally, we translated the classification of each pixel according to the PLS-DA models into probabilistic images for easier interpretation.

As our models were strongly dependent on the standard deviation of the different sequences applied to the selected ROIs, we calculated the apparent standard deviation for each pixel. This apparent standard deviation was calculated from the pixel selected and the neighbouring pixels in all directions. For statistical precision, the T1 signal intensity of weighted images, and T2 and T2* relaxation times for each pixel was substituted by the mean of the 4 pixels used for the calculation of the standard deviation. To calculate the probability of each pixel belonging to either the infarcted or the remote area, we ran all the pixels of each slice for each sample through the corresponding acute or chronic PLS-DA model. The PLS-DA model provided the information for RGB probabilistic maps. The probability of each pixel being classified as infarcted or remote corresponded to the relative intensity of the R or B channels respectively. The G channel was suppressed to avoid interference. The new RGB 3D matrix was flattened and overlaid in order to obtain the parametric map, providing a quantitative, pixel-by-pixel classification of the cardiac tissue.

## Supplementary information

Supplementary Material
