# Supplementary material for: Magnetic resonance microscopy and correlative histopathology of the infarcted heart
Source: Sci Rep. 2019 Dec 27;9:20017. doi: 10.1038/s41598-019-56436-5 (PMC6934559; doi:10.1038/s41598-019-56436-5)
Supplement: Supplementary file 1 — Supplementary Material [file 41598_2019_56436_MOESM1_ESM.docx]

**Magnetic resonance microscopy and correlative histopathology of the infarcted heart**

Itziar Perez-Terol MSc^1^, Cesar Rios-Navarro MSc^2^, Elena de Dios MSc^2^, Jose M. Morales PhD^1,3,4^, Jose Gavara MSc^2^, Nerea Perez-Sole BSc^2^, Ana Diaz PhD^3^, Gema Minana MD PhD^2,5,6^, Remedios Segura-Sabater PhD^1^, Clara Bonanad MD PhD^2,6^, Antoni Bayés-Genis MD PhD^5,7^, Oliver Husser MD PhD^8^, Jose V. Monmeneu MD PhD^9^, Maria P. Lopez-Lereu MD PhD^9^, Julio Nunez MD PhD^2,5,6^, Francisco J. Chorro MD PhD^2,5,6^, Amparo Ruiz-Sauri MD PhD^4^, Vicente Bodi* MD PhD^2,5,6^, Daniel Monleon* PhD^1,4,10^

^1^Laboratory of Metabolomics. Institute of Health Research-INCLIVA. Valencia, Spain.

^2^Department of Cardiology. Hospital Clinico Universitario. INCLIVA. Valencia. Spain.

^3^Unidad Central de Investigación Biomédica. University of Valencia, Valencia, Spain.

^4^Pathology Department, School of Medicine, University of Valencia. Valencia, Spain.

^5^Centro de Investigación Biomédica en Red – Cardiovascular (CIBER-CV).

^6^Medicine Department, School of Medicine, University of Valencia. Valencia, Spain.

^7^Cardiology Department and Heart Failure Unit, Hospital Universitari Germans Trias i Pujol. Department of Medicine, Universitat Autònoma de Barcelona, Barcelona, Spain.

^8^Department of Cardiology, St.-Johannes-Hospital, Dortmund, Germany.

^9^Cardiovascular Magnetic Resonance Unit. ERESA. Valencia. Spain.

^10^Centro de Investigación Biomédica en Red – Fragilidad y Envejecimiento Saludable (CIBER-FES). Madrid, Spain.

***Address for correspondence:**

Vicente Bodi MD, PhD. Department of Cardiology, Hospital Clinico Universitario-CIBERCV. INCLIVA. University of Valencia. Blasco Ibañez 17, 46010, Valencia, Spain. Telephone number: +34-96-3862600. Fax: +34-96-1973979. E-mail: [vicente.bodi@uv.es](mailto:vicente.bodi@uv.es)

***Co-corresponding author:**

Daniel Monleon PhD. Laboratory of Metabolomics, INCLIVA. University of Valencia. Menendez Pelayo 4acc, 46010, Valencia, Spain. Tel: +34 963864145. E-mail: daniel.monleon@uv.es

**SUPPLEMENTARY FIGURE**

**Figure S1. Histopathology criteria to differentiate oedema, inflammatory infiltration, and necrosis.**

Captures at 20X of haematoxylin-eosin stains from infarcted myocardial tissue isolated from 1-week reperfusion (acute myocardial infarction) group were selected to illustrate oedema (a), inflammatory infiltration (b), and necrosis (c). Oedema is characterized by liquid accumulation between cells; infiltration is defined as a region with a massive accumulation of leukocytes present; areas with disintegrated cells are considered necrotic tissue.


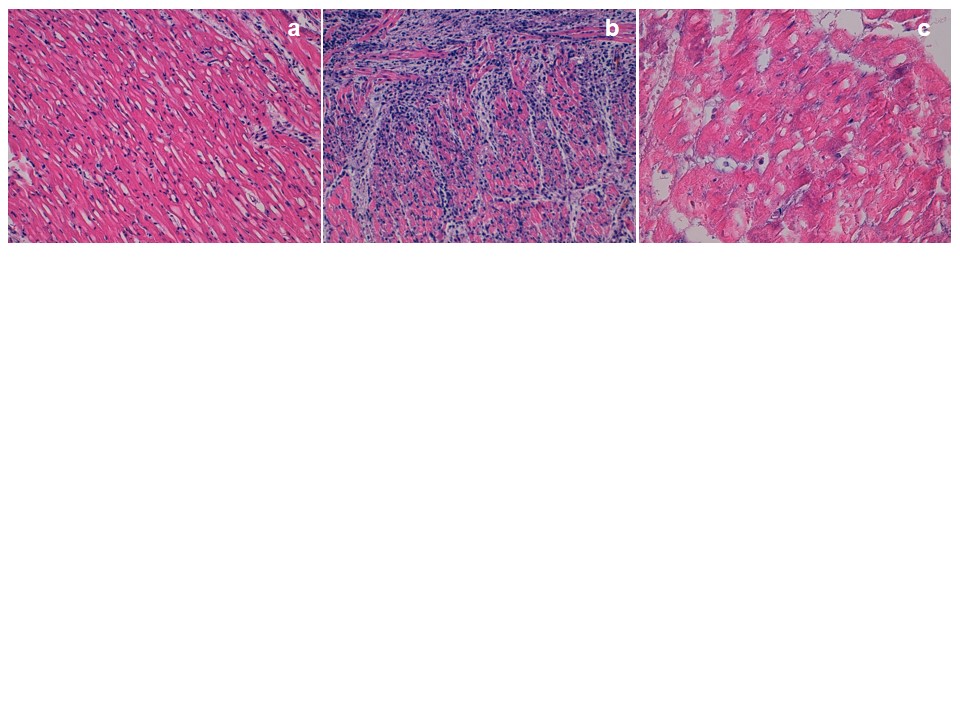


**SUPPLEMENTARY TABLES**

**Table S1.** P-Values obtained in the ANOVA analysis from the parameters determined using the magnetic resonance sequences in the three experimental groups.

|  | **ACUTE MI GROUP** | | | | | |
| --- | --- | --- | --- | --- | --- | --- |
| **p-values** | **REMOTE** | | | **INFARCT** | | |
|  | **T1 signal intensity** | **T2 map** | **T2* map** | **T1 signal intensity** | **T2 map** | **T2* map** |
| **CONTROL** | 3.30x10^-39^ | 0.0096 | 0.0114 | 0.0114 | 5.44x10^-14^ | 5.30x10^-11^ |
| **INFARCT** | 1.73x10^-9^ | 7.68x10^-8^ | 7.68x10^-8^ |  |  |  |

|  | **CHRONIC MI GROUP** | | | | | |
| --- | --- | --- | --- | --- | --- | --- |
| **p-values** | **REMOTE** | | | **INFARCT** | | |
|  | **T1 signal intensity** | **T2 map** | **T2* map** | **T1 signal intensity** | **T2 map** | **T2* map** |
| **CONTROL** | 4.49x10^-25^ | 9.13x10^-8^ | 5.43x10^-31^ | 1.10x10^-22^ | 0.0003 | 2.21x10^-23^ |
| **INFARCT** | 0.3822 | 2.82x10^-18^ | 2.82x10^-18^ |  |  |  |

**Abbreviation**: MI=myocardial infarction.

**Table S2.** Signal-noise-ratio for all chronic samples used in the study.

| **CHRONIC MI GROUP** | |  |  | **SNR T1_weighted images** | **SNR T2 map_signal intensity** | **SNR T2* map_signal intensity** |
| --- | --- | --- | --- | --- | --- | --- |
| **INFARCT** | SAMPLE 1 | AGAROSE ROI | Slice 1 | 95.14 | 97.91 | 141.10 |
|  |  |  | Slice 2 | 156.94 | 115.05 | 177.16 |
|  |  | TISSUE ROI | Slice 1 | 156.82 | 111.48 | 163.64 |
|  |  |  | Slice 2 | 236.78 | 127.50 | 193.91 |
|  | SAMPLE 2 | AGAROSE ROI | Slice 1 | 105.81 | 304.48 | 184.39 |
|  |  |  | Slice 2 | 46.18 | 245.30 | 149.48 |
|  |  | TISSUE ROI | Slice 1 | 129.57 | 323.64 | 195.40 |
|  |  |  | Slice 2 | 60.44 | 246.52 | 175.18 |
|  | SAMPLE 3 | AGAROSE ROI | Slice 1 | 195.48 | 175.62 | 168.08 |
|  |  |  | Slice 2 | 188.75 | 152.89 | 117.85 |
|  |  | TISSUE ROI | Slice 1 | 212.85 | 174.08 | 147.48 |
|  |  |  | Slice 2 | 216.52 | 167.60 | 113.97 |
|  | SAMPLE 4 | AGAROSE ROI | Slice 1 | 126.39 | 107.66 | 290.72 |
|  |  |  | Slice 2 | 131.25 | 162.88 | 328.53 |
|  |  | TISSUE ROI | Slice 1 | 147.20 | 124.24 | 313.52 |
|  |  |  | Slice 2 | 148.25 | 175.75 | 343.50 |
|  | SAMPLE 5 | AGAROSE ROI | Slice 1 | 110.70 | 68.84 | 171.93 |
|  |  |  | Slice 2 | 131.70 | 99.24 | 182.43 |
|  |  | TISSUE ROI | Slice 1 | 137.60 | 74.11 | 177.36 |
|  |  |  | Slice 2 | 164.77 | 105.66 | 188.37 |
|  | SAMPLE 6 | AGAROSE ROI | Slice 1 | 112.30 | 141.18 | 169.59 |
|  |  |  | Slice 2 | 103.75 | 110.05 | 243.56 |
|  |  | TISSUE ROI | Slice 1 | 126.50 | 125.67 | 153.02 |
|  |  |  | Slice 2 | 114.22 | 102.77 | 235.25 |
|  | SAMPLE 7 | AGAROSE ROI | Slice 1 | 128.89 | 109.26 | 169.88 |
|  |  |  | Slice 2 | 119.10 | 135.29 | 109.53 |
|  |  | TISSUE ROI | Slice 1 | 137.44 | 98.15 | 170.52 |
|  |  |  | Slice 2 | 122.97 | 118.09 | 110.91 |
| **REMOTE** | SAMPLE 1 | AGAROSE ROI | Slice 1 | 89.43 | 174.57 | 47.16 |
|  |  |  | Slice 2 | 106.13 | 109.19 | 55.86 |
|  |  | TISSUE ROI | Slice 1 | 85.65 | 117.29 | 44.18 |
|  |  |  | Slice 2 | 93.65 | 73.90 | 47.21 |
|  | SAMPLE 2 | AGAROSE ROI | Slice 1 | 124.72 | 126.17 | 157.00 |
|  |  |  | Slice 2 | 136.13 | 231.38 | 157.93 |
|  |  | TISSUE ROI | Slice 1 | 110.96 | 89.74 | 135.08 |
|  |  |  | Slice 2 | 118.04 | 156.78 | 131.87 |
|  | SAMPLE 3 | AGAROSE ROI | Slice 1 | 154.56 | 241.96 | 151.41 |
|  |  |  | Slice 2 | 185.30 | 169.37 | 184.34 |
|  |  | TISSUE ROI | Slice 1 | 173.52 | 164.50 | 145.46 |
|  |  |  | Slice 2 | 140.36 | 96.37 | 127.18 |
|  | SAMPLE 4 | AGAROSE ROI | Slice 1 | 132.66 | 24.98 | 23.14 |
|  |  |  | Slice 2 | 118.93 | 30.59 | 33.03 |
|  |  | TISSUE ROI | Slice 1 | 133.86 | 26.52 | 23.30 |
|  |  |  | Slice 2 | 111.94 | 29.70 | 32.50 |
|  | SAMPLE 5 | AGAROSE ROI | Slice 1 | 153.42 | 23.25 | 34.46 |
|  |  |  | Slice 2 | 166.20 | 18.30 | 54.77 |
|  |  | TISSUE ROI | Slice 1 | 170.01 | 21.41 | 33.45 |
|  |  |  | Slice 2 | 189.45 | 18.51 | 54.09 |
|  | SAMPLE 6 | AGAROSE ROI | Slice 1 | 106.98 | 109.64 | 184.94 |
|  |  |  | Slice 2 | 111.17 | 97.75 | 177.87 |
|  |  | TISSUE ROI | Slice 1 | 157.90 | 97.31 | 179.04 |
|  |  |  | Slice 2 | 178.84 | 90.57 | 181.21 |
|  | SAMPLE 7 | AGAROSE ROI | Slice 1 | 150.58 | 116.64 | 285.78 |
|  |  |  | Slice 2 | 141.27 | 57.07 | 154.58 |
|  |  | TISSUE ROI | Slice 1 | 137.33 | 85.32 | 337.03 |
|  |  |  | Slice 2 | 135.23 | 45.46 | 187.28 |

**Abbreviation**: ROI=region of interest.

**Table S3.** Signal-noise-ratio (SNR) for all acute samples used in the study.

| **ACUTE MI GROUP** | |  |  | **SNR T1_weighted images** | **SNR T2 map_signal intensity** | **SNR T2* map_signal intensity** |
| --- | --- | --- | --- | --- | --- | --- |
| **INFARCT** | **SAMPLE 1** | AGAROSE ROI | Slice 1 | 109.91 | 248.11 | 156.30 |
|  |  |  | Slice 2 | 100.71 | 262.96 | 200.08 |
|  |  | TISSUE ROI | Slice 1 | 198.15 | 230.54 | 199.92 |
|  |  |  | Slice 2 | 182.64 | 257.24 | 263.11 |
|  | **SAMPLE 2** | AGAROSE ROI | Slice 1 | 126.30 | 322.84 | 266.55 |
|  |  |  | Slice 2 | 112.50 | 284.89 | 287.50 |
|  |  | TISSUE ROI | Slice 1 | 150.79 | 291.38 | 238.81 |
|  |  |  | Slice 2 | 142.44 | 254.51 | 269.07 |
|  | **SAMPLE 3** | AGAROSE ROI | Slice 1 | 140.14 | 180.84 | 238.57 |
|  |  |  | Slice 2 | 134.28 | 177.75 | 194.75 |
|  |  | TISSUE ROI | Slice 1 | 194.14 | 260.03 | 260.26 |
|  |  |  | Slice 2 | 183.75 | 192.59 | 196.44 |
|  | **SAMPLE 4** | AGAROSE ROI | Slice 1 | 101.34 | 292.49 | 259.56 |
|  |  |  | Slice 2 | 102.01 | 312.42 | 219.55 |
|  |  | TISSUE ROI | Slice 1 | 150.24 | 323.11 | 238.66 |
|  |  |  | Slice 2 | 152.37 | 326.34 | 215.46 |
|  | **SAMPLE 5** | AGAROSE ROI | Slice 1 | 96.01 | 234.50 | 133.03 |
|  |  |  | Slice 2 | 81.34 | 228.32 | 115.31 |
|  |  | TISSUE ROI | Slice 1 | 137.77 | 257.21 | 130.00 |
|  |  |  | Slice 2 | 145.34 | 293.23 | 126.54 |
|  | **SAMPLE 6** | AGAROSE ROI | Slice 1 | 83.09 | 364.07 | 143.32 |
|  |  |  | Slice 2 | 91.62 | 317.28 | 206.60 |
|  |  | TISSUE ROI | Slice 1 | 148.46 | 412.87 | 181.80 |
|  |  |  | Slice 2 | 149.25 | 334.36 | 248.53 |
|  | **SAMPLE 7** | AGAROSE ROI | Slice 1 | 151.25 | 298.32 | 199.09 |
|  |  |  | Slice 2 | 138.30 | 316.25 | 210.65 |
|  |  | TISSUE ROI | Slice 1 | 167.74 | 300.83 | 170.33 |
|  |  |  | Slice 2 | 146.96 | 328.87 | 177.53 |
|  | **SAMPLE 8** | AGAROSE ROI | Slice 1 | 138.90 | 329.46 | 375.51 |
|  |  |  | Slice 2 | 107.69 | 285.70 | 329.76 |
|  |  | TISSUE ROI | Slice 1 | 156.05 | 254.39 | 336.98 |
|  |  |  | Slice 2 | 133.87 | 230.83 | 320.10 |
|  | **SAMPLE 9** | AGAROSE ROI | Slice 1 | 150.73 | 267.67 | 228.22 |
|  |  |  | Slice 2 | 110.27 | 278.14 | 297.33 |
|  |  | TISSUE ROI | Slice 1 | 172.16 | 243.55 | 245.53 |
|  |  |  | Slice 2 | 134.96 | 247.86 | 315.90 |
| **REMOTE** | **SAMPLE 1** | AGAROSE ROI | Slice 1 | 83.66 | 207.49 | 177.95 |
|  |  |  | Slice 2 | 162.81 | 249.07 | 194.04 |
|  |  | TISSUE ROI | Slice 1 | 148.94 | 206.10 | 207.92 |
|  |  |  | Slice 2 | 259.92 | 223.00 | 214.32 |
|  | **SAMPLE 2** | AGAROSE ROI | Slice 1 | 103.99 | 234.42 | 188.00 |
|  |  |  | Slice 2 | 87.59 | 286.62 | 204.31 |
|  |  | TISSUE ROI | Slice 1 | 103.99 | 183.49 | 191.35 |
|  |  |  | Slice 2 | 137.71 | 238.65 | 228.47 |
|  | **SAMPLE 3** | AGAROSE ROI | Slice 1 | 90.09 | 229.76 | 231.85 |
|  |  |  | Slice 2 | 77.93 | 189.21 | 186.54 |
|  |  | TISSUE ROI | Slice 1 | 178.81 | 264.31 | 299.10 |
|  |  |  | Slice 2 | 146.00 | 222.03 | 233.91 |
|  | **SAMPLE 4** | AGAROSE ROI | Slice 1 | 68.70 | 340.739546 | 165.957841 |
|  |  |  | Slice 2 | 58.60 | 235.773585 | 172.4411 |
|  |  | TISSUE ROI | Slice 1 | 101.34 | 300.282914 | 175.524544 |
|  |  |  | Slice 2 | 86.348 | 211.88776 | 180.848988 |
|  | **SAMPLE 5** | AGAROSE ROI | Slice 1 | 94.69 | 299.91 | 252.10 |
|  |  |  | Slice 2 | 89.25 | 249.01 | 232.06 |
|  |  | TISSUE ROI | Slice 1 | 137.56 | 264.08 | 277.924 |
|  |  |  | Slice 2 | 137.64 | 238.55 | 261.43 |

**Abbreviation**: ROI=region of interest.

**SUPPLEMENTARY MATERIAL**

The various characteristics of infarction under cardiac magnetic resonance are the result of the fundamental magnetic properties of human tissue. These characteristics reflect the outcome of reperfusion therapy and are important for prognosis in survivors of ST-segment elevation myocardial infarction ^1^. The longitudinal relaxation time of tissue (T1) is sensitive to the free water and to the proteins that interact with the water. T1 is longer when the water content (free and bound water both) is greater ^2^. T1 is mainly related to tissue fibrosis, oedema, and the ratio of cellular to extracellular space. The native T1 values increase in pathologies with high extracellular volume. The transverse relaxation time of tissue (T2) is sensitive to the volume of free water, and provides a quantitative measure of myocardial oedema ^3,4^. Finally, effective transversal relaxation (T2*) mapping is generally used to quantify myocardial iron content, and in the context of acute myocardial infarction can also delineate haemorrhage in the infarct core ^5^ because it is linked to blood oxygenation ^6^. Despite the benefits provided by endogenous contrast T1, T2 and T2* tissue parameters, it is gadolinium-enhanced cardiac magnetic resonance that has become a routine tool in post-infarction imaging, although its use is not entirely without risk.

A PRESS location sequence without presaturation was applied in a voxel of 2 cm^3^ of water and cardiac tissue at room temperature and with the 10 mm coil for microimage probe. The PRESS sequence consists of one 90˚ pulse, then two 180˚pulses applied orthogonally to obtain the signal from only one voxel. This sequence is equivalent to the pulse-acquired sequence but has echoes. Using the PRESS sequence, the full width at half maximum (FWHM) value for the water was 22.34 Hz in contrast to the 35.6 Hz for the cardiac tissue. In addition, we have a pulse-acquired sequence released in a water tub with a FWHM of 19 Hz as an optimal data reference in a homogeneous sample.

**REFERENCES**

1. Carrick, D. *et al*. Pathophysiology of LV remodelling in survivors of STEMI: Inflammation, remote myocardium, and prognosis. *JACC Cardiovasc. Imaging* **8**, 779-789 (2015).
2. Michel, E., Hernandez, D. & Lee, S. Y. Electrical conductivity and permittivity maps of brain tissues derived from water content based on T1‐weighted acquisition. *Magn. Reson. Med.* **77**, 1094-1103 (2017).
3. Roller, F. C., Harth, S., Schneider, C. & Krombach, G. A. T1, T2 Mapping and extracellular volume fraction (ECV): application, value and further perspectives in myocardial inflammation and cardiomyopathies. *Fortschr. Röntgenstr.* **187**, 760-770 (2015).
4. Ferreira, V., Piechnik, S., Robson, M., Neubauer, S. & Karamitsos, T. Myocardial tissue characterization by magnetic resonance imaging novel applications of T1 and T2 mapping. *J. Thorac. Imaging* **29**, 147-154 (2014).
5. Baritussio, A., Scatteia, A. & Bucciarelli-Ducci, C. Role of cardiovascular magnetic resonance in acute and chronic ischemic heart disease. *Int. J. Cardiovasc. Imaging* **33**, 1-14 (2017).
6. Melbourne, A. *et al*. Separating fetal and maternal placenta circulations using multiparametric MRI. *Magn. Reson. Med.***81**, 350-361 (2019).
